# The impact of discharge readiness on post-traumatic growth in patients after thyroid cancer surgery: the mediating role of sickness-related stigma

**DOI:** 10.3389/fonc.2024.1361036

**Published:** 2024-09-02

**Authors:** Bin Huang, Guangzhi Liu, Jiaqian Huang, Susu He, Wen Li, Shanshan Xiao, Xiaohua Song, Hongtao Chen

**Affiliations:** ^1^ School of Nursing, Hunan University of Chinese Medicine, Changsha, China; ^2^ Xiangya Hospital of Central South University, Central South University, Changsha, Hunan, China; ^3^ Hunan Cancer Hospital/The Affiliated Cancer Hospital of Xiangya School of Medicine, Central South University, Changsha, Hunan, China

**Keywords:** post-operative thyroid cancer, readiness for discharge, post-traumatic growth, stigma, mediating role

## Abstract

**Objective:**

To investigate the relationship between post-traumatic growth, morbidity stigma and readiness for discharge in post-operative thyroid cancer patients.

**Methods:**

422 post-operative thyroid cancer patients from three tertiary care hospitals in Hunan and Tianjin were surveyed using the General Information Questionnaire, the Post-traumatic Growth Scale, the Readiness for Discharge Scale, and the Social Influence Scale.

**Results:**

Discharge readiness positively predicted the level of post-traumatic growth in thyroid cancer patients (P < 0.01), and morbidity stigma negatively predicted post-traumatic growth (P < 0.01), with morbidity stigma playing a mediated role between discharge readiness and post-traumatic growth.

**Conclusions:**

Readiness for discharge can positively predict post-traumatic growth, and morbidity stigma plays a mediating role between readiness for discharge and post-traumatic growth. It is suggested that clinical and nursing staff should strengthen patients’ discharge readiness guidance and education, help patients and their families establish an effective feedback mechanism for disease condition and psychological cognitive condition, focus on reducing patients’ sense of shame, and improve patients’ physical and mental health.

## 1 Introduction

Thyroid cancer is the most common endocrine malignancy, with a rapidly increasing incidence rate. It has become one of the top ten prevalent malignant tumors and ranks among the top five in terms of incidence rates in the female population (1). Surgical treatment is currently an effective method for thyroid cancer; however, post-operative patients may experience physical and psychological complications such as voice loss, depression, and hypothyroidism (2). Post-operative patients may confront stigma associated with fears of cancer recurrence, cancer-related fatigue, and societal marginalization, collectively contributing to a deterioration in their quality of life (3, 4). Traditional research has primarily focused on the negative emotional impacts on patients. With the development of positive psychology, the positive effects brought about by stressors like cancer are increasingly recognized. Studies have pointed out that while experiencing negative trauma, cancer patients can also perceive the positive and beneficial aspects, which promote self-management and improve quality of life (Holland et al., 2010). This phenomenon is referred to as post-traumatic growth. Post-traumatic growth is a significant factor influencing patients’ social adaptability and recovery compliance. A higher level of post-traumatic growth plays a crucial role in improving patient’s quality of life, strengthening psychological function, and enhancing life awareness.

Discharge readiness is a patients’ perception of their own preparedness for discharge, encompassing physical stability, adequate support, psychological capacity, sufficient information, and knowledge. It also entails a comprehensive analysis by healthcare professionals of the patient’s physical and mental health status to assess whether they are adequately prepared for discharge. Accurate assessment of discharge readiness can help reduce unplanned readmission rates and minimize the occurrence of adverse events. Several studies indicate that satisfactory discharge readiness can promote post-traumatic growth and facilitate timely recovery for patients (5, 6).

Sickness-related stigma refers to a sense of shame experienced internally due to discrimination and prejudice caused by stereotypes associated with diseases such as thyroid cancer. This stigma arises from the inherent bias towards cancer and physical trauma suffered by patients, leading to discrimination and isolation by others. Based on the Conservation of Resources Theory, both adequate internal and external resources contribute positively to patients’ adaptive responses to negative experiences, promoting a positive transformation. The theory of social support emphasizes the organic connection between individuals and society. Individuals acquire tools, information, companionship, emotional support, and evaluations from their social support networks. These resources play a significant supportive role for individuals in negative states, promoting better mood maintenance and overall health (7, 8). Therefore, as a positive individual resource and support, discharge readiness is likely to assist in resisting the resource exhaustion brought about by the negative experience of sickness-related stigma, thereby inducing a positive psychological transformation. Sickness-related stigma might serve as a mediator between discharge readiness and post-traumatic growth. When facing stressors such as cancer and surgical trauma, discharge readiness provides patients with internal resources, enhancing individual coping abilities, manifesting stronger adaptability and promoting post-traumatic growth. Given the lack of exploration into the mechanism behind the relationship between discharge readiness and post-traumatic growth in foreign literature, this research selects patients after thyroid cancer surgery as subjects to elucidate the mediating mechanisms through which discharge readiness promotes post-traumatic growth. It aims to provide theoretical and empirical support to clinical practices striving to improve patients’ quality of life, offering valuable decision-making references and practical rationale for promoting patients’ physical and mental recovery, improving patients’ quality of life, and facilitating the formulation of relevant clinical interventions.

## 2 Methods

### 2.1 Study subjects

The study encompassed a questionnaire survey carried out among patients who had undergone thyroid cancer surgery and met the inclusion criteria in three Grade A tertiary hospitals in Hunan and Tianjin from February to September 2022.

Inclusion Criteria:

(1) Patients who were diagnosed with thyroid cancer and had undergone thyroidectomy; the evaluation of clinical symptoms and signs, imaging examinations, fine-needle aspiration cytology (FNAC), preoperative serum thyroid tumor marker detection [such as thyroglobulin (Tg) and calcitonin (Ca-125)], and postoperative pathological examination are in line with the diagnostic criteria for thyroid cancer (9).
(2) Aged between 18-75 years old;
(3) Possessing sufficient reading and writing abilities;
(4) Those who voluntarily participated in the survey.

Exclusion Criteria:

(1) A history of psychiatric sickness, severe cardiovascular, cerebrovascular, renal diseases, or other medical conditions;
(2) Communicatively impaired individuals;
(3) Patients with recurrence after thyroid cancer surgery;
(4) Patients with tumors in other parts of the body.

### 2.2 Research tools

#### 2.2.1 General Information Survey

This survey form includes general demographic data and disease-related information. The demographic data comprises variables such as gender, age, religious beliefs, residential location, marital status, education level, occupation, etc. The disease-related information encompasses details about the presence or absence of adjunctive treatment, type of thyroid cancer, and post-operative emotional state of the patient.

#### 2.2.2 Post-traumatic Growth Scale

The Post-traumatic Growth Scale is used to measure the extent of positive change experienced by individuals following traumatic events. The original scale was developed by Tedeschi et al. (10) based on a literature review of human responses to highly stressful events and interviews with individuals who had experienced spousal bereavement, physical disability, and other life crises. The scale used in this study (11) has 5 dimensions and 21 items: Personal Strength (4 items), New Possibilities (5 items), Appreciation of Life (3 items), Relationships with Others (7 items), and Spiritual Change (2 items). Each item is scored on a scale from 0 to 5, with a total score ranging from 0 to 105. A higher total score on the scale indicates a greater degree of positive change and growth following negative events. The internal consistency coefficient for the total scale is 0.90, while the internal consistency coefficients for each dimension range from 0.67 to 0.85. In this study, the Cronbach’s α coefficient for the scale is 0.831.

#### 2.2.3 Discharge Readiness Assessment Scale

This scale (12) comprises three dimensions and consists of twelve items. The dimensions include “Personal Status,” composed of three items, “Anticipatory Support,” with four items, and “Adaptability,” incorporating five items. Each item is positively scored within a range of 0 to 10. The total score for the scale ranges from 0 to 120, with higher scores indicating a greater level of readiness for discharge. Conversely, lower total scores suggest an elevated risk of encountering post-discharge adaptation challenges and limited access to support. In this research study, the Cronbach’s α coefficient for the scale was calculated as 0.819.

#### 2.2.4 Social Impact Scale

Fife and Wright (13), drawing upon Link’s modified labeling theory (14, 15), conceptualized and constructed a scale to quantitatively assess sickness-related stigma. Originally designed for measuring the impact of sickness-related stigma on self-concept among cancer and HIV/AIDS patients, this scale offers a distinctive focus on evaluating the social ramifications of stigmatization, thereby holding considerable relevance for patient adaptation and clinical practice. In comparison to other existing scales that primarily measure sickness-related stigma, such as the devaluation-discrimination scale, the Social Impact Scale provides a more comprehensive examination of the broader societal implications arising from stigmatization. The utilized scale in this study comprises 24 items, systematically addressing four distinct dimensions: social rejection, economic insecurity, internalized shame, and social isolation. To ensure comprehensive coverage, all items have been reverse-scored utilizing a 4-point rating scale. The total score ranges from 0 to 96, with higher scores reflecting heightened perceptions of social impact and stronger manifestations of sickness-related stigma. The Cronbach’s α coefficient for the scale ranges from 0.85 to 0.90, and the correlation coefficients for each dimension range from 0.28 to 0.66. Currently, this scale is widely implemented for measuring sickness-related stigma among patients with chronic diseases such as cancer. In this study, the Cronbach’s α coefficient for the scale is 0.953.

### 2.3 Data collection

Preceding the survey, an extensive training and guidance session was conducted to ensure uniformity among the surveyors. Prior approvals were obtained from the participating hospitals, while informed consent was acquired from all participants. A standardized script was employed to introduce the survey process and elucidate its objectives to the respondents, ensuring consistency in information dissemination. The principles of confidentiality were thoroughly elucidated to the participants, emphasizing the utmost importance of safeguarding their personal information. The data collection process encompassed a combination of in-person, face-to-face administration of questionnaires on-site, as well as the utilization of an online platform (e.g., Wenjuanxing) to facilitate data gathering. This multifaceted approach ensured flexibility and accessibility for participants, while also maintaining data integrity and security.” Following the completion of questionnaire collection, any questionnaires found to contain significant omissions or random responses were deemed invalid and subsequently excluded from the analysis. The remaining valid questionnaires were assigned unique codes for identification purposes, and a double-check procedure was implemented during data entry using Excel 2016 to establish the primary database. Additional logical checks were conducted to ensure the exclusion of any lingering invalid questionnaires. In this particular study, a total of 470 questionnaires were distributed, with 422 valid questionnaires ultimately collected, yielding a commendable valid response rate of 89.8%.

### 2.4 Statistical methods

The analysis of data was conducted utilizing the SPSS version 26.0 statistical software. Descriptive statistics, including frequencies and percentages, were employed to summarize categorical variables. To assess group comparisons, the Mann-Whitney U test, a non-parametric statistical test for independent samples, was employed. Structural equation modeling was utilized to analyze the mediating effects, and significance of regression coefficients was assessed using the Bootstrap method. Statistical significance was defined as a p-value less than 0.05 (P<0.05).

## 3 Results

### 3.1 General characteristics of post-thyroid cancer surgery patients

A total of 422 individuals who underwent thyroid cancer surgery were enrolled in this study, with a mean age of (42.88 ± 10.82) years. In terms of residential location, the majority of participants (333 patients; 78.9%) resided in urban areas, while a smaller proportion (89 patients; 21.1%) lived in rural areas. With regard to education level, 19 patients (4.5%) had completed primary school, 77 patients (18.2%) had completed junior high school, 81 patients (19.2%) had completed senior high school or vocational school, and 155 patients (36.7%) had attained a college degree or higher level of education. In terms of marital status, 40 patients (9.5%) were unmarried, 361 patients (85.5%) were married, 12 patients (2.8%) were divorced, and 9 patients (2.1%) were widowed. With respect to living arrangements, 31 patients (7.3%) resided alone, whereas the majority of participants, comprising 391 patients (92.7%), lived with others. Among the participants included in this study, the distribution of thyroid cancer types was as follows: 327 patients (77.5%) were diagnosed with papillary carcinoma, 4 patients (0.9%) had follicular carcinoma, 5 patients (1.2%) presented with undifferentiated carcinoma, 3 patients (0.5%) had medullary carcinoma, and 83 patients (19.7%) had an uncertain type of thyroid cancer. In terms of post-operative emotions, 51 patients (12.1%) reported experiencing significant mood swings and irritability, 53 patients (12.6%) indicated feeling happier during mood fluctuations, 90 patients (21.3%) expressed anger during these episodes, while the majority of participants, 227 patients (53.8%), reported relatively stable emotions without frequent outbursts. The tumor staging of the patients is as follows: Stage I cancer: 104 patients (24.6%), Stage II cancer: 141 patients (33.5%), Stage III cancer: 155 patients (36.7%), Stage IV cancer: 22 patients (5.2%). Please refer to **Table 1** for detailed information.

**Table 1:** Partial general demographic information (n=422).

	Variable	Count (%)
Residence	Urban residents	333 (78.9%)
	Rural residents	89 (21.1%)
Education level	Primary education	19 (4.5%)
	Middle school education	77 (18.2%)
	High school or vocational school education	81 (19.2%)
	College degree or higher education	155 (36.7%)
Marital status	Single	40 (9.5%)
	Married	361 (85.5%)
	Divorced	12 (2.8%)
	Widowed	9 (2.1%)
Whether to live alone	Living alone	31 (7.3%)
	Living with others	391 (92.7%)
Cancer staging	Stage I cancer	104 (24.6%)
	Stage II cancer	141 (33.5%)
	Stage III cancer	155 (36.7%)
	Stage IV cancer	22 (5.2%)
Histological type	Papillary carcinoma	327 (77.5%)
	Follicular carcinoma	4 (0.9%)
	Undifferentiated carcinoma,	5 (1.2%)
	Medullary carcinoma	3 (0.7%)
	Uncertain type of thyroid cancer	83 (19.7%)
Motional state	Significant mood swings and irritability	51 (12.1%)
	Feeling happier during mood swings	53 (12.6%)
	Expressing anger during mood swings	90 (21.3%)
	Emotionally stable	227 (53.8%

### 3.2 Scores of post-traumatic growth, discharge readiness, and sickness-related stigma in patients following thyroid cancer surgery

The participants included in this study obtained an average score of (61.12 ± 11) points on the post-traumatic growth scale. The scores for each dimension were as follows: personal strength (13.16 ± 2.86), appreciation of life (8.7 ± 2.14), interpersonal relationships (20.8 ± 4.39), new possibilities (14.3 ± 3.79), and spiritual changes (4.15 ± 1.77). On the Discharge Readiness Scale, the participants achieved an average score of (85.59 ± 15.38) points, with scores for each dimension as follows: personal status (17.38 ± 5.74), adaptability (40.94 ± 8.56), and anticipatory support (30.72 ± 8.87). The sickness-related stigma score was (55.61 ± 12.21) points, with scores for social rejection (19.24 ± 5.52), economic insecurity (7.14 ± 1.80), internalized stigma (12.57 ± 2.68), and social isolation (16.53 ± 4.11) (**Table 2**).

**Table 2:** Post-traumatic growth, readiness for discharge, and stigma scores of patients after thyroid cancer surgery (n=422, $\bar{\chi }$ ± S).

Term	Score ( $\bar{\chi }\pm \text{S}$ )
Post-traumatic Growth	61.12 ± 11
Dimension of Personal Strength	13.16 ± 2.86
Dimension of Appreciation of Life	8.7 ± 2.14
Dimensions of Relation to others	20.8 ± 4.39
Dimension of New Possibilities	14.3 ± 3.79
Dimension of Spiritual Changes	4.15 ± 1.77
Discharge Readiness	85.59 ± 15.38
Personal Status	17.38 ± 5.74
Adaptability	40.94 ± 8.56
Anticipatory Support	30.72 ± 8.87
Stigma	55.61 ± 12.21
Social Rejection	19.24 ± 5.52
Economic Insecurity	7.14 ± 1.80
Internalized Stigma	12.57 ± 2.68
Social Isolation	16.53 ± 4.11

### 3.3 Correlation analysis of post-traumatic growth, discharge readiness, and sickness-related stigma in patients diagnosed with thyroid cancer

A correlation analysis was performed to examine the relationships between traumatic growth, discharge readiness, and sickness stigma in a sample of 422 post-operative thyroid cancer patients. The findings indicated statistically significant correlations among these variables. Specifically, there was a negative correlation between discharge readiness and sickness stigma (r=-0.300, p<0.01), a positive correlation between discharge readiness and post-traumatic growth (r=0.139, p<0.139), and a negative correlation between sickness stigma and post-traumatic growth (r=-0.180, p<0.01).

### 3.4 The mediating role of stigma in the association between discharge readiness and post-traumatic growth among patients diagnosed with thyroid cancer

**Figure 1:**
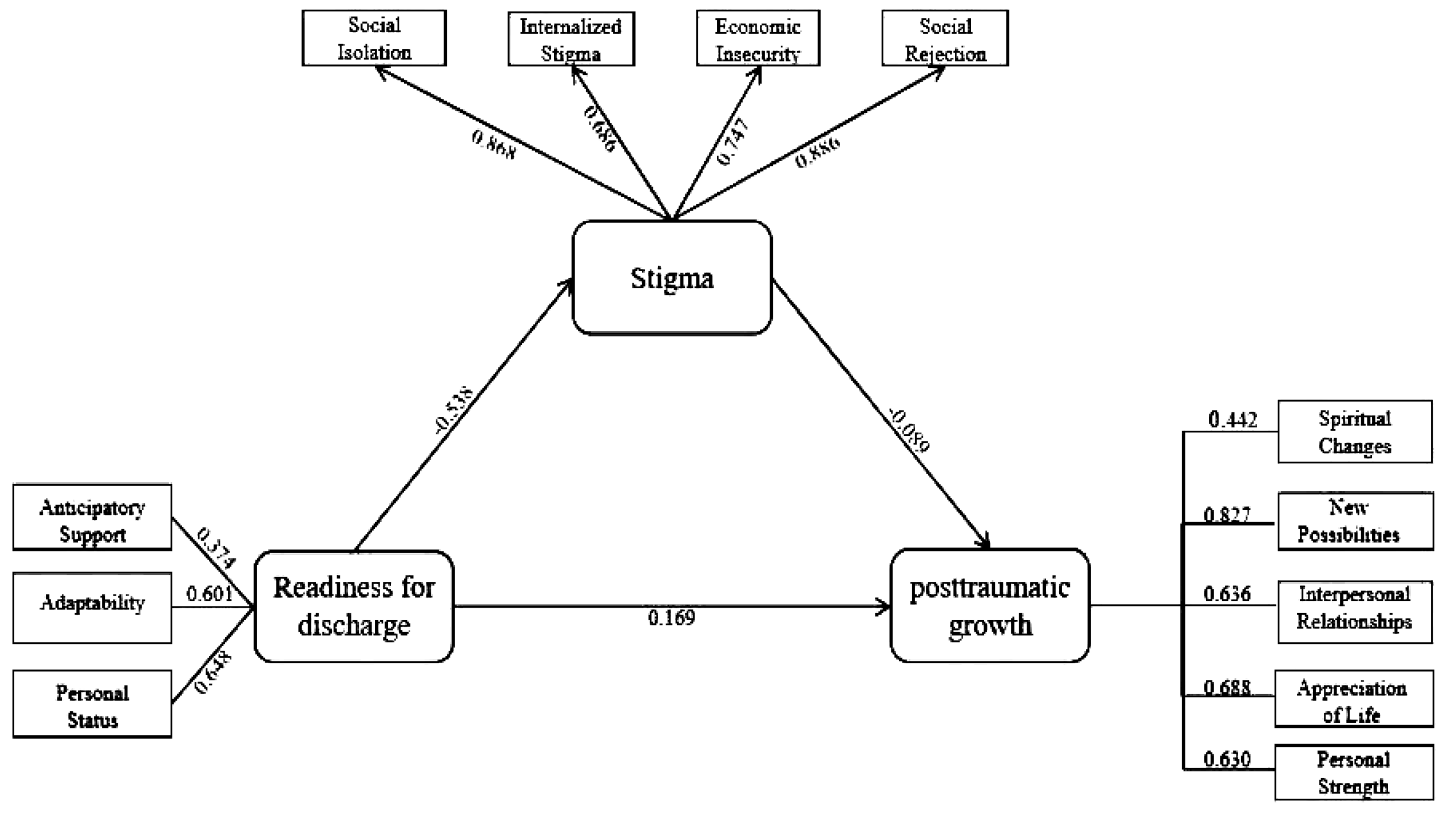
Structural equation model diagram (n=422).

The results of the Pearson correlation analysis indicated a significant positive correlation between discharge readiness and post-traumatic growth (r = 0.139, p < 0.01). Conversely, stigma showed a negative correlation with post-traumatic growth (r = -0.180, p < 0.01). Additionally, a negative correlation was observed between discharge readiness and stigma (r = -0.300, p < 0.01). The structural equation model demonstrated satisfactory fit indices: χ2/d ratio = 1.769, comparative fit index = 0.901, Tucker-Lewis index = 0.912, root mean square error of approximation = 0.131, non-normed fit index = 0.944, incremental fit index = 0.927, goodness-of-fit index = 0.939, and adjusted goodness-of-fit index = 0.927. These fit indices indicated that the model was acceptable. The structural diagram of the mediating effect of stigma is presented in **Figure 1**. The results of the mediation analysis indicated that discharge readiness had a negative indirect effect on post-traumatic growth through stigma (B=-0.238, p<0.001). Additionally, stigma exerted a negative indirect effect on post-traumatic growth (B=-0.136, p<0.001). Bootstrap resampling with 5000 iterations was conducted to test the mediating effect, and the results revealed a fully mediating model (c’ nonsignificant), indicating that the direct effect was not significant. This suggests that stigma plays a mediating role in the relationship between discharge readiness and post-traumatic growth (**Tables 3**, **4**).

**Table 3:** The mediating role of sickness stigma between readiness for discharge and post-traumatic growth (n=422).

Effect	Pathway	Coefficients B	SE	t	*p*	95%CI
Direct	Readiness → post-traumatic growth	0.099^**^	0.035	2.880	0.004	0.030~0.168
Indirect	Post-traumatic growth → stigma	-0.238^**^	0.037	-6.455	0.000	-0.311~-0.165
	Stigma → post-traumatic growth	-0.136^**^	0.045	-3.018	0.003	-0.224~-0.048
Total	Readiness → post-traumatic growth	0.067	0.036	1.867	0.063	-0.004~0.138

SE, standard error. ** p<0.01. A → B; means the path of the relationship between variable A and variable B.

**Table 4:** The total effect of sickness stigma.

Term	C (total effect)	a	b	C (direct effect)	a*b (mediating role)	conclusion
Total pathway	0.099^**^	-0.238^**^	-0.136^**^	0.067	0.033	Mediating Effects

Total pathway, Readiness - stigma - post-traumatic growth; ^**^P<0.01. *b represents the product of the effects of a and b.

## 4 Discussion

### 4.1 Postoperative patients with thyroid cancer exhibit moderate levels of post-traumatic growth and needs improvement

Effective discharge readiness, as a positive resource, significantly reduces the stigma associated with illness. This study confirms that discharge readiness directly influences the stigma experienced by postoperative thyroid cancer patients. Head and neck cancer patients often suffer from anxiety, stress, and loneliness, and the physical trauma and societal discrimination related to cancer can exacerbate the stigma felt by these patients post-thyroidectomy (16, 17). Reducing this stigma requires psychological and material support from healthcare providers and family members, enhancing patients’ understanding of the disease, and fostering positive self-perception. A high level of discharge readiness indicates that patients can mitigate stigma through better self-perception and social support. Given the favorable prognosis and longer survival time of postoperative thyroid cancer patients, their discharge readiness is often overlooked. Enhancing discharge readiness is essential for promoting patients’ physical and mental health.

Moreover, discharge readiness, as a positive material and psychological resource, may aid patients in cognitive restructuring and foster positive psychological changes, known as post-traumatic growth, following the adverse event of cancer and surgery (18). The promotion of post-traumatic growth by discharge readiness can be attributed to several factors: (1) Comprehensive discharge preparation, including physical stabilization, adequate information and knowledge provision, sufficient support, and psychological capability development. When patients are in good overall health, adaptable, and expect support, they exhibit positive beliefs about their illness and high health literacy, facilitating multidimensional self-management and promoting post-traumatic growth, consistent with Cieslak et al. (19). (2) Studies on discharge readiness indicate that it impacts readmission rates and the implementation of secondary prevention measures during hospitalization (20). Efficient discharge preparation within a limited time can encourage patients to actively change their lifestyles, eliminating risk factors that may lead to thyroid cancer recurrence. Thus, healthcare providers should establish effective feedback mechanisms. By discussing patients’ conditions and psychological mechanisms with them and their families, healthcare providers can identify knowledge gaps and develop effective educational measures to improve adherence to treatment recommendations. Strengthening communication among interdisciplinary teams and encouraging patient and family feedback can help identify their needs, implement personalized services, reduce readmission risk, and promote post-traumatic growth. Comparing our research findings with previous studies on trauma patients, our Pearson correlation coefficient is lower (21, 22). This discrepancy may be due to differences in disease types or regional population differences. Additionally, it is essential to consider that this study was conducted in the unique context of the COVID-19 pandemic, which adds complexity. There may be certain moderating variables between discharge readiness and post-traumatic growth. However, the levels of these moderating variables may have changed due to the evolving nature of the pandemic, leading to a lower correlation between discharge readiness and post-traumatic growth compared to previous studies. Future researchers should further explore these aspects to better understand the potential influencing factors between discharge readiness and post-traumatic growth in postoperative thyroid cancer patients.

The level of post-traumatic growth in postoperative thyroid cancer patients is slightly higher than in previous studies (23), possibly due to regional and temporal factors, with the investigators in this study likely receiving more support from family and healthcare providers. Post-traumatic growth in postoperative thyroid cancer patients remains at a moderate level, with room for further improvement. The lowest-scoring dimension in this study is “new possibilities,” indicating a need to enhance patients’ hope and life perception. Some patients experience fluctuating negative emotions, hindering their ability to maintain a calm mindset and cognitively process adverse events (24). This negative emotional state may impair their ability to gain new insights and recognize opportunities for personal growth and development. Additionally, other scholars have pointed out (25, 26) that when discussing surgical outcomes with patients, healthcare providers can share successful peer cases, offer targeted support and interventions (27), and boost patients’ confidence in their recovery process, enabling them to approach their environment with a calm mindset, ultimately promoting their post-traumatic growth. It is recommended that healthcare providers advocate the benefits of spousal support, give more attention to divorced, widowed, and single-living patients, encourage patients to share experiences, foster common interests, and promote physical exercise to reduce cancer-related loneliness (28, 29), as these groups showed lower levels of post-traumatic growth in this study.

### 4.2 Mediating role of sickness stigma in the relationship between discharge readiness and post-traumatic growth

This study indicates that disease-related shame plays a mediating role in the relationship between discharge readiness and post-traumatic growth. There is a correlation between shame and post-traumatic growth (21). Previous research on post-thyroidectomy patients has been limited, and this study also confirms the correlation between discharge readiness in post-thyroidectomy patients and both post-traumatic growth and shame. From the perspective of the conservation of resources theory, resources determine how individuals cope with stressful situations. When existing resources are at risk of loss or depletion, individuals experience stress and seek resources to offset the loss (30). When facing stressors such as cancer and surgical trauma, discharge readiness can provide internal resources for patients, helping them to counter the resource depletion caused by disease-related shame, enhance their coping abilities, and promote adaptive capacity and post-traumatic growth. This study validates this model and identifies shame as an intermediate process of resource consumption. The research results indicate that the level of discharge readiness is negatively correlated with the level of trauma. This indicates that the internal and external resource reserves of patients at discharge may have a potential impact on their positive psychological transformation postoperatively. The results indicate that disease-related shame plays a mediating role between discharge readiness and post-traumatic growth. This suggests that shame is related to the level of post-traumatic growth and plays an important role in the impact of discharge readiness. In particular, in post-thyroidectomy patients, due to the relatively good prognosis, medical staff and family members often overlook the assessment of patients’ discharge readiness, which is not conducive to the accumulation of the patient’s internal and external resources. When their level of discharge readiness is low, shame related to the disease may consume positive inner resources, and negative cognition and coping strategies may exacerbate, which is not conducive to positive transformation of the inner self.

Therefore, it is recommended that medical staff and family members closely monitor the level of shame in patients, and promptly ascertain whether patients feel discriminated against and self-doubt due to the shame brought about by cancer. Nursing staff can actively promote physical exercises, including brisk walking, square dancing, jogging, and aerobic exercises to enhance muscle strength (such as sit-ups and push-ups). Traditional Chinese exercises such as Tai Chi, Baduanjin, and martial arts can stimulate the release of endorphins in the brain, alleviate negative emotions, and restore a normal psychological state. With the rise of continuous care, medical staff can provide medical interventions through methods such as telephone follow-ups, WeChat platforms, home visits, and home care services to ensure continuous and collaborative medical interventions in different medical environments.

## 5 Limitations and future directions

Our survey results need to carefully consider several inherent limitations in order to interpret them appropriately. Due to the cross-sectional nature of the current survey, there may be errors in determining the causal relationship between discharge readiness and post-traumatic growth in post-thyroidectomy patients. It is suggested that in future investigations, researchers employ experimental or longitudinal survey methods. For example, using methods such as repeated measurements, trajectory models, or cross-lagged models. These methods allow for repeated measurements of post-traumatic growth levels in post-thyroidectomy patients and can observe transformations over a longer period of time. By utilizing these methods, researchers can gain a more thorough and profound understanding and arrive at more definitive findings. Furthermore, it is worth noting that the sampling process may exhibit a certain degree of bias, mainly because the survey questionnaire used is typically targeted at post-thyroidectomy patients accessible within the provinces of Hunan and Tianjin. Subsequent surveys can increase the sample size of post-thyroidectomy patients by consciously including participants from various regions, incorporating international multicenter surveys, and employing more precise sampling methods such as stratified sampling to mitigate inherent potential biases in the sampling process, thereby ensuring its applicability and universality.

## 6 Conclusion

The post-traumatic growth levels among post-operative thyroid cancer patients were found to be moderate, with room for improvement. Discharge readiness and sickness stigma were identified as crucial influencing factors for post-traumatic growth, with sickness stigma playing a mediating role between discharge readiness and post-traumatic growth. These findings highlight the need for clinical healthcare professionals to strengthen discharge readiness guidance and education for patients. Effective feedback mechanisms regarding patients’ illness conditions and psychological perceptions can be established with patients and their families. Furthermore, developing more specific measurement tools based on objective assessments will aid in identifying patients’ knowledge gaps and formulating effective educational interventions. In addition, healthcare professionals can implement intervention measures such as traditional Chinese ear acupuncture, acupuncture therapy, and narrative nursing to reduce patients’ sickness stigma. Continuity of care can also be facilitated through post-discharge follow-up via platforms like WeChat and telephone consultations. These interventions aim to promote post-traumatic growth and mitigate the risk factors associated with thyroid cancer recurrence among patients.

## Data Availability

The raw data supporting the conclusions of this article will be made available by the authors, without undue reservation.

## References

[1] Filetti S Durante C Hartl D Leboulleux S Locati LD Newbold K. Thyroid cancer: ESMO Clinical Practice Guidelines for diagnosis, treatment and follow-updagger. Ann Oncol. (2019) 30:1856–83. doi: 10.1093/annonc/mdz400 31549998

[2] Wang C Chen X Wei X Chen F Wang Y Shen Z. Recurrence factors and prevention of complications of pediatric differentiated thyroid cancer. Asian J Surg. (2017) 40:55–60. doi: 10.1016/j.asjsur.2016.09.001 27697309

[3] Williamson TJ Garon EB Shapiro JR Chavira DA Goldman JW Stanton AL. Facets of stigma, self-compassion, and health-related adjustment to lung cancer: A longitudinal study. Health Psychol. (2022) 41:301–10. doi: 10.1037/hea0001156 PMC9030259 35324247

[4] Hamann HA Williamson TJ Studts JL Ostroff JS. Lung cancer stigma then and now: continued challenges amid a landscape of progress. J Thorac Oncol. (2021) 16:17–20. doi: 10.1016/j.jtho.2020.10.017 33384057 PMC8020298

[5] Galvin EC Wills T Coffey A. Readiness for hospital discharge: A concept analysis. J Adv Nurs. (2017) 73:2547–57. doi: 10.1111/jan.13324 28440958

[6] Wang Y Li J Zhai M Zhao Y Li Q. Exploring readiness for discharge, quality of discharge teaching, and fear of disease progression in lung cancer patients undergoing chemotherapy: A correlation analysis. Thorac Cancer. (2023) 15:66–76. doi: 10.1111/1759-7714.15164 PMC10761620 37984977

[7] Price DM Unger Z Wu Y Meyers K Golub SA. Clinic-level strategies for mitigating structural and interpersonal HIV pre-exposure prophylaxis stigma. AIDS Patient Care STDS. (2022) 36:115–22. doi: 10.1089/apc.2021.0176 PMC8971970 35289691

[8] Moeini B Barati M Farhadian M Heydari Ara M. The effectiveness of an educational intervention to enhance happiness in Iranian older people: Applying social support theory. Australas J Ageing. (2020) 39:86–93. doi: 10.1111/ajag.12702 31325220

[9] Zauszniewski JA Eggenschwiler K Preechawong S Roberts BL Morris DL. Effects of teaching resourcefulness skills to elders. Aging Ment Health. (2006) 10:404–12. doi: 10.1080/13607860600638446 16798633

[10] Beck T Zhang N Shah A Khoncarly S McHenry C Jin J. Thyroid cancer identified after positron emission tomography (PET) shows aggressive histopathology. J Surg Res. (2004) 260:245–50. doi: 10.1016/j.jss.2020.11.012 33360690

[11] Wang J. Development of posttraumatic growth inventory and its norm for patient with accidental trauma. Shanghai(SH: Second Military Medical University (2011).

[12] Lin YH Kao CC Huang AM Chi MT Chou FH. Psychometric testing of the Chinese version of the Readiness for Hospital Discharge Scale. J Nurs. (2014) 61:56–65. doi: 10.6224/JN.61.4.56 25116315

[13] Fife BL Wright ER. The dimensionality of stigma: a comparison of its impact on the self of persons with HIV/AIDS and cancer. J Health Soc Behav. (2000) 41:50–67. doi: 10.2307/2676360 10750322

[14] Moses T. Self-labeling and its effects among adolescents diagnosed with mental disorders. Soc Sci Med. (2009) 68:570–8. doi: 10.1016/j.socscimed.2008.11.003 19084313

[15] Link BG Phelan JC. Conceptualizing stigma. Annu Rev Sociol. (2001) 27:363–85. doi: 10.1146/annurev.soc.27.1.363

[16] Park KA Kim S Oh EG Kim H Chang HS Kim SH. Factors affecting the health-promoting behavior of thyroid cancer survivors: comparison by stage of cancer survivorship. Support Care Cancer. (2022) 30:3429–39. doi: 10.1007/s00520-022-06799-9 PMC8857080 34999951

[17] Tseng WT Lee Y Hung CF Lin PY Chien CY Chuang HC. Stigma, depression, and anxiety among patients with head and neck cancer. Support Care Cancer. (2022) 30:1529–37. doi: 10.1007/s00520-021-06550-w 34533631

[18] Park CL. Making sense of the meaning literature: An integrative review of meaning making and its effects on adjustment to stressful life events. Psychol Bull. (2010) 136:257–301. doi: 10.1037/a0018301 20192563

[19] Cieslak R Benight C Schmidt N Luszczynska A Curtin E Clark RA. Predicting posttraumaticgrowth among Huricane Katrina Kissinger, P.survivors living with HIV: the role of self-efficacy, social support and PTSD symptoms. Anxiety Stress 2 Coping. (2009) 22:449–03. doi: 10.1080/10615800802403815 19296264

[20] Lau E Adams YJ Ghiaseddin R Sobiech K Ehla EE. Discharge readiness and associated factors among postpartum women in tamale, Ghana. West J Nurs Res. (2023) 45:539–46. doi: 10.1177/01939459231152122 36782383

[21] Hu R Wang X Liu Z Hou J Liu Y Tu J. Stigma, depression, and post-traumatic growth among Chinese stroke survivors: A longitudinal study examining patterns and correlations. Top Stroke Rehabil. (2022) 29:16–29. doi: 10.1080/10749357.2020.1864965 33371827

[22] Wei W Li X Tu X Zhao J Zhao G. Perceived social support, hopefulness, and emotional regulations as mediators of the relationship between enacted stigma and post-traumatic growth among children affected by parental HIV/AIDS in rural China. AIDS Care. (2016) 28 Suppl 1:99–105. doi: 10.1080/09540121.2016.1146217 PMC4828627 26899475

[23] Lyons SJ. Stress reactions in the face of the” Good cancer”: correlates of posttraumatic growth in thyroid cancer. Canada: University of Toronto (2017).

[24] Growney CM English T. Age and cognitive ability predict emotion regulation strategy use. J Gerontol B Psychol Sci Soc Sci. (2023) 78:987–97. doi: 10.1093/geronb/gbad021 PMC10214650 36744761

[25] Yunhui X Hao GU Qing Z Dejie LI Ying G Junxing X. Efficacy of meridian massage for motor function after a stroke: a systematic review and Meta-analysis. J Tradit Chin Med. (2022) 42:321–31. doi: 10.19852/j.cnki.jtcm.2022.03.001 PMC9924753 35610001

[26] Zhang Y He X Hu S Hu S He F Shen Y. Efficacy and safety of massage in the treatment of post-stroke insomnia: A protocol for systematic review and meta-analysis. Med (Baltimore). (2020) 99:e23598. doi: 10.1097/MD.0000000000023598 PMC7748325 33371092

[27] Jerome L McNamee P Abdel-Halim N Elliot K Woods J. Solution-focused approaches in adult mental health research: A conceptual literature review and narrative synthesis. Front Psychiatry. (2023) 14:1068006. doi: 10.3389/fpsyt.2023.1068006 37065885 PMC10098109

[28] Jin R Xie T Zhang L Gong N Zhang J. Stigma and its influencing factors among breast cancer survivors in China: A cross-sectional study. Eur J Oncol Nurs. (2021) 52:101972. doi: 10.1016/j.ejon.2021.101972 33991869

[29] Yan MH Fan YY Zhang JE. Stigma, self-efficacy and late toxicities among Chinese nasopharyngeal carcinoma survivors. Eur J Cancer Care (Engl). (2022) 31:e13528. doi: 10.1111/ecc.13528 34668257

[30] Hobfoll SE. Conservation of resources theory: Its implication for stress, health, and resilience. Appl Psychol: Int Rev. (2011) 60:177–86. doi: 10.1080/1047840X.2015.1002377

